# Information Network Modeling for U.S. Banking Systemic Risk

**DOI:** 10.3390/e22111331

**Published:** 2020-11-23

**Authors:** Giancarlo Nicola, Paola Cerchiello, Tomaso Aste

**Affiliations:** 1 Department of Economics and Management, University of Pavia, 27100 Pavia, Italy; giancarlo.nicola01@universitadipavia.it (G.N.); paola.cerchiello@unipv.it (P.C.); 2 Department of Computer Science, University College London, London WC1E 6EA, UK; 3 Systemic Risk Centre, London School of Economics, London WC2A 2AE, UK

**Keywords:** granger causality, graphical models, financial stress

## Abstract

In this work we investigate whether information theory measures like mutual information and transfer entropy, extracted from a bank network, Granger cause financial stress indexes like LIBOR-OIS (London Interbank Offered Rate-Overnight Index Swap) spread, STLFSI (St. Louis Fed Financial Stress Index) and USD/CHF (USA Dollar/Swiss Franc) exchange rate. The information theory measures are extracted from a Gaussian Graphical Model constructed from daily stock time series of the top 74 listed US banks. The graphical model is calculated with a recently developed algorithm (LoGo) which provides very fast inference model that allows us to update the graphical model each market day. We therefore can generate daily time series of mutual information and transfer entropy for each bank of the network. The Granger causality between the bank related measures and the financial stress indexes is investigated with both standard Granger-causality and Partial Granger-causality conditioned on control measures representative of the general economy conditions.

## 1. Introduction

The stability of the financial system is a basic condition for sustainable growth of an economy as a whole. Its importance arises from the key role of financial institutions in capital allocation, that is, the transfer of financial resources from entities with surplus funds to entities with deficit funds. The 2008 crisis, triggered by large writedowns of bank assets related to subprime mortgages, unfortunately demonstrated such idea. The crisis was characterized by the bankruptcy or distress of several large banks like Bear Stearns, Citigroup, Lehman Brothers, Merrill Lynch, Wachovia, and Washington Mutual that in several cases, had to be rescued by the government. Such instability of the financial system resulted in a severe credit and liquidity crunch in the financial markets affecting the real economy. This type of risk, wherein the entire financial system is simultaneously distressed, is generally referred to as systemic risk. Systemic risk, when it occurs, impacts not only financial markets and institutions, but also the real economy as a whole due to decreases in capital supply and increases in capital costs.

The term systemic risk was coined in the early 1980s by the economist William Cline [1] at the onset of the Latin American debt crisis. According to his definition, systemic risk is a threat that disturbances in the financial system will have serious adverse effects on the entire financial market and on the real economy. Systemic risk models address the issue of interdependence between financial institutions and, specifically, measures how bank default risks are transmitted among banks [2,3].

The last few years have witnessed an increasing research literature on systemic risk, with the aim of identifying the most contagious institutions and their transmission channels. Specific measures of systemic risk have been proposed for the banking sector; in particular by Reference [4,5] (MES), Reference [6,7,8] (SRISK), Reference [9] (δCoVaR), Reference [10] (CES), and Reference [11]. These approaches leverage financial market price information to asses the financial institution’s risk from the estimated loss probability distribution, conditional on a crash event in the financial market. However, they do not address the issue of risk transmission between different banks. In order to address this aspect of systemic risk, researchers have introduced financial network models [12,13,14,15]. Networks have emerged as a useful tool for understanding contagion and systemic risk, in financial systems. In fact, after the 2008 financial crisis, there have been many studies on financial networks and their role in systemic risk. A major finding emphasized by these studies is that financial contagion is mainly driven by system-wide interconnectedness of institutions. In particular, Reference [16] propose several econometric measures of connectedness based on Granger-causality networks and principal component analysis. References [17,18] propose tail dependence network models aimed at overcoming the bivariate nature of the available systemic risk measures. Systemic risk models are typically based on the assumption of full connectedness among all nodes, which makes their interpretation difficult and also their estimation hard when a large number of them is being considered.

In tackling this limitation, Reference [19] proposes LASSO regularized Vector Autoregressive models for selecting the significant links in a network model. Information filtering networks models have been applied to socio-economic and financial systems for a long time starting form the pioneering work of Reference [20] on the hierarchical structure of financial markets via minimum spanning tree and then expanding beyond trees by References [21,22]. Graphical correlation models, which can account for partial connectedness, expressed in terms of conditional independence constraints have been used by [23,24]. A similar but alternative approach has been explored by Reference [25] introducing multivariate Brownian processes with a correlation structure determined by a conditional independence graph.

Correlation networks have proven to be a suitable tool to visualize the structure of pairwise marginal correlations among a set of nodes corresponding to the investigated banking systems. In these models each bank is represented by a node in the network and each pair of nodes can be connected by an edge, which has a weight related to the correlation coefficient between the two nodes. Furthermore, the banking system represented with these models can be described by the adjacency and inverse covariance matrix of the corresponding graphical model. The LoGo approach by Reference [26] provides a way to generate a probabilistic graphical model from information filtering networks. LoGo is a valid alternative to LASSO characterized by a very meaningful network structure, with a computationally efficient and fast inference, that allows us to update the graphical model each market day. With these daily updates of the graphical models, we can generate daily time series of mutual information and transfer entropy for the system and for each bank of the network from April 2003 to May 2017. Our contribution follows this latter development estimating a Graphical Gaussian Model on the market prices of the 74 largest listed U.S. banks using LoGo.

We then investigate how the information theory measures (mutual information and transfer entropy) extracted from the estimated graphical models, correlate with and Granger cause financial stress indexes like LIBOR-OIS spread, STLFSI and USD/CHF exchange rate. The rationale behind is to understand how these measures compare to the financial stress indexes and which banks show Granger causality links with the indexes. The Granger causality between the bank related measures and the financial stress indexes is investigated applying Partial Granger-causality tests conditioning on control measures representative of the general economy conditions.

We will leverage the dynamic ‘snapshots’ of how the U.S. bank system stock correlations evolve to generate several time series of measures extracted from the network model. In fact, it is possible to extract different bank-related quantities from the graphical model (namely mutual information, page rank, transfer entropy, number of bank edges) that, highlighting different properties, allow for an inspection of the system evolution.

The remainder of this paper is organized as follows. In Section 2 we discuss the general methodology introducing graphical models and their theoretical background. Then we briefly recall the LoGo methodology that we use to infer the graphical model on the bank network. In Section 2.2 we introduce the measures that we calculate from the bank network model, the Granger causality test and in particular the partial Granger causality test and discuss their use. In Section 3 we present the bank stocks data that we use to fit the network model and the financial stress indexes whose causality relationship is investigated. In Section 4 we present the results of the causality analysis between the financial stress indexes and the measures extracted from the bank stock network model and finally in Section 5 we briefly discuss and recap the results of the paper.

## 2. Methodology

The approach we propose in this paper is multistage: first we fit a graphical model based on the LoGo algorithm to infer the structure of significant interconnections among banks, afterwords we calculate graph based measures, namely Mutual Information and Cross Entropy to be used for further investigations.

While the system aggregated measures give us information regarding the overall system financial stress through bank level measures, we would like to investigate which banks help to predict the financial stress indexes. The bank level timeseries are computed aggregating the single edges mutual information and transfer entropy in the way explained in Section 2.2 for each market day. After this process we have three measures relative to each single bank:

- Mutual information (the total mutual information between the bank and its neighbours);
- Transfer entropy inflow (the sum of the transfer entropy incoming to the bank from its 1-day lagged neighbours);
- transfer entropy outflow (the sum of the transfer entropy going from the 1-day lagged bank node to its contemporary neighbours).

This three measures summarize, respectively, three types of information; (i) how much a bank returns are correlated with the rest of the banking system (more precisely with its neighbours); (ii) how much knowing a bank returns helps predicting the rest of the system returns; (iii) how much knowing the system returns helps to predict a bank returns. We want to investigate whether some of these specific bank measures helps in predicting general financial stress indexes like STLFSI, Libor-OIS spread and USD/CHF exchange rate. From such a finding we could understand that a bank has an important role in the financial stress dynamics of the system. To test if these measures help to predict the financial stress indexes, we resort to the Granger causality test and in particular to a recent improvement of it, the partial Granger causality [27]. We chose the partial Granger causality to mitigate the possible confounding influence in the eventuality of missing and latent variables [28] as explained below. Indeed, when testing for causality, we also condition on three macroeconomic variables to control the effect of the macroeconomic cycle and eventual spurious correlations. These three control variables are: the 10Y US Treasuries yield, the gold price and the EUR/USD exchange ratio and are related to the general economic conjuncture. For the partial Granger causality test we resort to the R package FIAR (Functional Integration Analysis in R) [29]. We test the linear partial Granger causality from the bank level timeseries to the different financial stress indexes for three different periods in which we split the study: pre-crisis (2003–2006), financial crisis (2007–2010), post-crisis (2011–2017). Prior to testing for causality, the timeseries have been normalized and a Dickey-Fuller test has been performed and, where necessary, the time series have been differentiated with R forecast package [30,31]. Each causality test is performed considering up to the 5th lags at the bank level time series.

### 2.1. Graphical Network Model

Statistics and social sciences in general have witnessed the proposal and employment of many techniques for network modeling. They can be summarized according to the following classes of models: exponential random graph models ([32,33]), stochastic block models ([34,35]) and latent space models ([36,37]). For a review of statistical models for social networks, see References [38,39,40,41]. In many applications, the network is typically assumed to be known and is considered as the observed data. However, taking such assumption in systemic risk modeling can be dangerous since the role of interconnectedness in the risk-propagation process crucially depends on the network structure, which is generally unknown. Much of the earlier work on contagion has focused on interconnectedness arising from actual exposures among institutions, based on either balance sheet information or other financial market data. There is relatively little empirical work on the former, largely because of problems of balance sheet data accessibility. However, several studies have focused on the latter in order to understand sources of contagion and spillovers ([16,19,25,42]). Market data are easy to gather and employ, however, understanding the most appropriate networks structure is an uncertain and complex task.

Bayesian approach to network identification takes into account network uncertainty by allowing us to incorporate prior information, where necessary, and perform model averaging (see Reference [43]). The approach is closely related to the literature on Gaussian graphical models for time series ([44,45,46] ). It is also related to References [47,48], who present the network techniques as a valid alternative to the Granger concept for causal identification and its extensions in the econometrics literature ([49,50]).

Here, we briefly describe the graphical network models that will be used to estimate relationships between the *N* banks, by means of market data. Direct relationships among banks can be measured by their partial correlations, that express the direct influence of one bank onto another one. Partial correlations can be estimated assuming that the observations follow a graphical Gaussian model, in which the covariance matrix Σ is constrained by the conditional independences described by a graph (see e.g., Reference [51]). More formally, let $X=\left(X_{1},...,X_{N}\right)\in R^{N}$ be a N−dimensional random vector distributed according to a multivariate normal distribution $N\left(\mu ,\Sigma \right)$. We will assume that data are generated by a stationary process therefore, without loss of generality, μ=0. In addition, we will assume throughout that the covariance matrix Σ is not singular.

Let G=(V,E) be an undirected graph, with vertex set $V=\left\{1,...,N\right\}$, and edge set E=V×V, a binary matrix, with elements $e_{ij}$, that describe whether pairs of vertices are (symmetrically) linked between each other ($e_{ij}=1$), or not ($e_{ij}=0$). If the vertices *V* of this graph are put in correspondence with the random variables $\left(X_{1},...,X_{N}\right)$, the edge set *E* is associated with conditional independence on *X* via the so-called Markov properties [51]. In particular, the pairwise Markov property determined by *G* states that, for all 1≤i<j≤N:

(1) $$e_{ij}=0⟺X_{i}⊥X_{j}|X_{V\backslash \{i,j\}};$$

that is, the absence of an edge between vertices *i* and *j* is 3 equivalent to independence between the random variables $X_{i}$ and $X_{j}$, conditionally on all other variables $X_{V\backslash \{i,j\}}$.

Let the elements of $\Sigma^{-1}$, the inverse of the covariance matrix, be indicated as $\left\{\sigma^{ij}\right\}$. Whittaker (1990) proved that the following equivalence also holds:

(2) $$X_{i}⊥X_{j}|X_{V\backslash \{i,j\}}⟺\rho_{ijV}=0,$$

where

(3) $$\rho_{ijV}=-\frac{\sigma^{ij}}{\sqrt{\sigma^{ii}\sigma^{jj}}}$$

denotes the ij-th partial correlation, that is, the correlation between $X_{i}$ and $X_{j}$, conditionally on the remaining variables $X_{V\backslash \{i,j\}}$.

Therefore, by means of the pairwise Markov property, and given an undirected graph G=(V,E), a graphical Gaussian model can be defined as the family of all *N*-variate normal distributions that satisfy the constraints induced by the graph on the partial correlations, as follows:

(4) $$e_{ij}=0⟺\rho_{ijV}=0$$

for all 1≤i<j≤N.

In our study we investigate a relatively large number of banks (74) and we take advantage of a recently presented algorithm LoGo [26] to estimate graphical models on the basis of time series data. LoGo is a methodology that makes use of information filtering networks to produce probabilistic models that are sparse and with high likelihood. One of its main advantages is that it is computationally fast, making possible applications with very large data sets. The LoGo algoritm calculates the global sparse inverse covariance matrix from a simple sum of local inverse covariances computed on small subparts of the network matrices. The use of low-dimensional local inversions makes the procedure computationally efficient, statistically robust and only slightly sensitive to the curse of dimensionality [26]. In particular the method is based on a recent, new family of information filtering networks, the triangulated maximal planar graph (TMFG) [52] that are decomposable graphs. A decomposable graph has the property that every cycle of length greater than three has a chord, an edge that connects two vertices of the cycle in a smaller cycle of length three. The construction of the algorithm, through a sum of local inversion, makes this methodology particularly suitable for parallel computing and dynamical adaptation by local, partial updating, as described in Reference [26] where a more detailed explanation of the method is presented.

### 2.2. Information Theory Measures

Since we build a Gaussian Graphical Model (GGM) for every market day, based on the stock time series of the 90 previous market days, we can calculate, every market day, the bank and system’s related measures that are representative of the previous 90 days trends. We obtain a time series from March 2003 to October 2017 for each measure that we calculate from the network model. We selected a time frame of 90 days to fit the graphical model for three main reasons: i. have more datapoints (90) than the number of banks (74); ii. Obtain a network representative only of the last few months; iii. the LoGo algorithm outperforms the Glasso specially in the case when the number of variables (banks) and datapoint (days) are comparable [26]. While it is possible to extract many interesting quantities from the network model like bank and system average partial correlations, number of edges, Pagerank and others, we focus on two quantities derived from information theory: Mutual Information and Transfer Entropy between banks.

The mutual information is a measure of the mutual dependence between the two variables and it quantifies the amount of information that one variable can tell us about the other. Intuitively, it measures the information shared by the two variables and quantifies how much knowing one variable reduces the uncertainty about the other one [53]. When two variables are independent, knowing one does not give any information about the other one and vice versa, their mutual information is zero. At the other extreme, if one variable is a deterministic function of the other and vice versa then all information conveyed by one variable is shared with the other one: knowing just one of them determines the value of the other one and vice versa. As a result, in this case the mutual information is the same as the uncertainty contained in one variable, namely their entropy. In general, if we represent the different entropies of the two random variables with an analogy to set theory, the mutual information is the intersection of the two sets and represents the uncertainty they have in common.

The definition of the mutual information for two continuous random variables *X* and *Y* is:

(5) $$I\left(X;Y\right)=\int_{Y}^{}\int_{X}^{}p\left(x,y\right)\log\left(\frac{p(x,y)}{p(x)p(y)}\right)dxdy,$$

where p(X,Y) is the joint probability density function of the two random variables, and p(X) and p(Y) are the marginal probability density functions of *X* and *Y* respectively. Mutual information however says little about causal relationships, because it lacks directional and dynamical information. In fact, it is symmetric between the random variables and thus, it cannot distinguish between driver and response variables [54].

Transfer Entropy (TE) is instead a measure of the amount of directed (time-asymmetric) transfer of information between the two variables. Thus, transfer entropy from a random variable X to another random variable Y is the amount of uncertainty reduced in future values of Y by knowing the past values of X given past values of Y [55]. In other words, transfer entropy is the conditional mutual information, with the history of the influenced variable $Y_{t-1:t-L}$ in the condition [56]

(6) $$TE_{X\to Y}=I\left(Y_{t};X_{t-1:t-L}|Y_{t-1:t-L}\right).$$

This means that the transfer entropy can be taken as an indicator to understand which are the driver and response variables in a system [57].

In our study, we calculate each day the mutual information and the transfer entropy among the banks of the network to produce a corresponding time series for each bank. The mutual information is updated every market day for every edge of the network (GGM inferred by the LoGo algorithm) and then for each bank we take the sum of the mutual information over its network edges. In other words, we obtain the sum of mutual information between a bank and all its direct neighbours. As a result, we obtain a measure for each bank that is the total mutual information of a bank given the set of neighbours; this time series describes the evolution of the mutual information between the bank and the rest of the banking system.

Similarly, we obtain a time series for the transfer entropy but in this case the time dimension is needed thus, the GGM fitting process based on the LoGo algorithm considers also one period lagged variables (lag−1), since the transfer entropy exists between lagged and contemporary variables as shown in Formula (6).

Consequently, every bank in the network is represented by a contemporary variable (e.g., $JPM_{t}$) and a lag-1 variable (e.g., $JPM_{t-1}$). For every bank couple (e.g., JPM and BAC) we have two directions of “transfer entropies”: $JPM_{t-1}$ to $BAC_{t}$ and viceversa $BAC_{t-1}$ to $JPM_{t}$. The former ($JPM_{t-1}$ to $BAC_{t}$) is a transfer entropy inflow for BAC and a transfer entropy outflow for JPM while the latter ($BAC_{t-1}$ to $JPM_{t}$) is a transfer entropy inflow for JPM and transfer entropy outflow for BAC. Thus, we have two different transfer entropy measures for each bank: “transfer entropy inflow” and “transfer entropy outflow”. As for the mutual information, for each bank we take the sum of these quantities over its edges (the bank first neighbours). The transfer entropy inflow is related to how much a bank stock behaviour is predictable given the previous behaviour of its neighbours while the outflow is related to how much a bank stock previous behaviour is useful for predicting its neighbours stocks. For sake of clearness and to ease the reader comprehension, we report in the Appendix A the pseudo-code in Algorithms A1–A3 tables that describe the steps applied to calculate respectively the mutual information, transfer entropy outflow and transfer entropy inflow.

### 2.3. Granger Causality

Since our research hypothesis aims at analyzing whether information theory measures extracted from the bank network are useful to predict financial stress indexes and if they cause them according to a temporal dimension, we need a method to assess such effect. In the following paragraph, we introduce the Granger causality test [58]. Granger causality entails the statistical notion of causality based on the relative forecast power of two time series. Time series *j* is said to “Granger-cause” time series *i* if past values of *j* contain information that helps predicting *i* above and beyond the information contained in past values of *i* alone. In a well known paper [58], Granger has proposed a useful test based on the following principle: if lagged values of a time series $X_{t}$ contribute to foresee current values of a time series $Y_{t}$ in a forecast achieved with lagged values of both $X_{t}$ and $Y_{t}$, then we say $X_{t}$ *Granger causes* $Y_{t}$. As was first shown in Reference [59], the Granger causality corresponds to the concept of exogeneity and it is therefore necessary to have an unidirectional causality in order to guarantee consistent estimation of distributed lag models. The mathematical formulation of this test is based on linear regressions of $X_{t+1}$ on $X_{t}$ and $Y_{t}$.

Since economic network models typically involve the step of ’structural model selection’, in which a relevant set of variables is selected for analysis, it is likely to exclude some relevant variables, which can lead to the detection of apparent causal interactions that are actually spurious [28]. A way to take into account such issue is by means of ’partial Granger causality’ introduced in Reference [27] together with some properties about its distribution. The idea is that latent variables may give rise to detectable correlations among the residuals of the corresponding vector AR model. By analogy with the concept of partial correlation [60], an additional term based on these correlations can mitigate the confounding influence of the latent variables.

Thus, in this paper, we prefer to employ the linear partial Granger causality test defined as follows. Considering 2 time series $X_{t}$ and $Z_{t}$ which admit a joint autoregressive representation as follows:

(7) $$X_{t}=\sum_{i=1}^{p}a_{1i}X_{t-i}+\sum_{i=1}^{p}c_{1i}Z_{t-i}+\varepsilon_{1t}$$

(8) $$Z_{t}=\sum_{i=1}^{p}b_{1i}Z_{t-i}+\sum_{i=1}^{p}d_{1i}X_{t-i}+\varepsilon_{2t}.$$

The variance-covariance matrix for the model can be represented as follows:

(9) $$S=\left[\begin{array}{cc} var(\varepsilon_{1t}) & cov(\varepsilon_{1t},\varepsilon_{2t})\\ cov(\varepsilon_{2t},\varepsilon_{1t}) & var(\varepsilon_{2t}). \end{array}\right]$$

Extending this concept further, the vector autoregressive representation for a system involving 3 times series and testing whether time series $Y_{t}$ Granger causes $X_{t}$ eliminating the effects of $Z_{t}$, we get the following:

(10) $$X_{t}=\sum_{i=1}^{p}a_{i}X_{t-i}+\sum_{i=1}^{p}s_{i}Y_{t-i}+\sum_{i=1}^{p}c_{i}Z_{t-i}+\varepsilon_{3t}$$

(11) $$Y_{t}=\sum_{i=1}^{p}e_{i}Y_{t-i}+\sum_{i=1}^{p}f_{i}X_{t-i}+\sum_{i=1}^{p}g_{i}Z_{t-i}+\varepsilon_{4t}$$

(12) $$Z_{t}=\sum_{i=1}^{p}b_{i}Z_{t-i}+\sum_{i=1}^{p}d_{i}X_{t-i}+\sum_{i=1}^{p}l_{i}Y_{t-i}+\varepsilon_{5t}.$$

The noise covariance matrix for the model can be represented as

(13) $$\Sigma =\left[\begin{array}{ccc} var(\varepsilon_{3t}) & cov(\varepsilon_{3t},\varepsilon_{4t}) & cov(\varepsilon_{3t},\varepsilon_{5t})\\ cov(\varepsilon_{4t},\varepsilon_{3t}) & var(\varepsilon_{4t}) & cov(\varepsilon_{4t},\varepsilon_{5t})\\ cov(\varepsilon_{5t},\varepsilon_{3t}) & cov(\varepsilon_{5t},\varepsilon_{4t}) & var(\varepsilon_{5t}). \end{array}\right]$$

The partial Granger causality can be calculated by selecting the necessary elements from the noise covariance matrices *S* and Σ in the following way

(14) $$F=\log\left(\frac{var\left(\varepsilon_{1t}\right)-\frac{cov{\left(\varepsilon_{1t},\varepsilon_{2t}\right)}^{2}}{var(\varepsilon_{2t})}}{var\left(\varepsilon_{3t}\right)-\frac{cov{\left(\varepsilon_{3t},\varepsilon_{5t}\right)}^{2}}{var(\varepsilon_{5t})}}\right).$$

The reader can easily notice that a relationship holds between partial Granger causality *F* and Transfer Entropy TE, that is: $TE=\frac{1}{2}F$.

While in theory, partial Granger causality is only able to eliminate confounders effects when their infuence is identical for every time series, however in Reference [61] it has been shown to be robust for deviations from this assumption. Moreover, in the presence of unknown latent and exogenous influences, it is shown in Reference [27] and again in Reference [61] that partial Granger causality better eliminates their influence than simple Granger causality.

### 2.4. Financial Indicators

For each bank, we consider the daily return obtained from the stock closing price of financial markets, for a period of 3716 days from January 2003 through October 2017, as follows:

(15) $$R_{t}=\ln\left(P_{t}/P_{t-1}\right),$$

where *t* is a day, t−1 the day preceding it and $P_{t}$, $P_{t-1}$ the corresponding closing prices of that bank in these days.

In our study, we inspect the causality relations among bank stocks and the overall system financial stress, thus we need to select some suitable stress indicators. To this aim, we consider three indexes commonly referred to when evaluating the stress of the financial system: the St. Louis Fed Financial Stress Index (STLFSI), the London Interbank Offering Rate–Overnight Index Swap spread (LIBOR-OIS spread) and USD/CHF exchange ratio.

The STLFSI is a financial stress index constructed by the Federal Reserve Bank of St. Louis. It measures the degree of financial stress in the markets and is constructed from 18 weekly data series: seven interest rate series, six yield spreads and five other indicators. STLFSI is built upon Principal Component Analysis method, in particular taking the first principal component of 18 distinct measures of financial stress and is thus a measure of overall financial market stress. The average value of the index, which begins in late 1993, is designed to be zero. Thus, zero is viewed as representing normal financial market conditions. Values below zero suggest below-average financial market stress, while values above zero suggest above-average financial market stress [62,63].

The LIBOR-OIS spread is the difference between the 3-month London Interbank Offered Rate (LIBOR) and the corresponding overnight indexed swap (OIS) rates and is regarded as a strong indicator of the health of the banking system [64]. The LIBOR is the interest rate at which banks borrow unsecured funds from other banks in the London wholesale money market for a period of 3 months. It is an important measure of risk and liquidity in the money market and thus an indicator for the relative stress in the money markets.

Compared to LIBOR, the LIBOR-OIS spread provides a more complete picture of how the market is viewing credit conditions because it strips out the effects of underlying interest-rate moves, which are in turn affected by factors such as central bank policy, inflation and growth expectations. During the financial crisis of 2007–2010, the LIBOR-OIS spread reached its maximum indicating a severe credit crunch and peaked concurrently with announcements of emergency funding to rescue Northern Rock, large write-downs by large investment banks and large bank failures.

The USD/CHF exchange rate is considered a measure of financial stress because in period of financial stress and instability safe haven inflows are likely to play a key role in the appreciation of the Swiss franc [65]. Currencies, in fact, can appreciate in times of crisis because they are offered as safe investment instruments by the countries issuing them. The currencies of such countries are commonly referred to as safe haven currencies and the media and the literature are unanimous in ascribing the strength of the Swiss franc to its status as a safe haven currency.

## 3. Data and Analysis Process

The data we analyze are bank stock price time series and for sake of comparability and homogeneity, we focus on a single banking market, the U.S. banking system. This is an interesting group of banks to study, due to its relevance in the world economy and particularly for its role in originating the 2008 financial crisis, with many large banks which have seriously impacted the world and U.S. economy and politics. We take into account the top 74 U.S. large listed banks, for which there exist daily financial market data that we have collected. In Table 1 on page 10, we report the list of the banks that we consider, along with their stock market code (ticker) and their total assets at the end of 2016 (in US Dollars).

In our study thus, we investigate the causality relationship among these three financial stress indicators and the measures extracted from the network model inferred with the LoGo algorithm from the bank stock time series. Moreover, when testing the Partial Granger Causality, we condition on three control variables related to the general economic conjuncture: 10Y US Treasuries yield, gold price and EUR/USD exchange ratio. We derived the time series of the daily returns for both the gold price and the EUR/USD exchange ratio (like for the case of the bank stocks) given by Equation (15) while the 10Y US Treasuries yield time series has been taken as it is due to its partially negative values and being already a yield. We decided to condition on these quantities in order to control for the effect of the general status of the economy in the partial Granger causality analysis.

Initially, we calculated a network of the U.S. banks over the entire time horizon (2003–2017) with the LoGo algorithm to have insights regarding the most correlated banks (from the stock price point of view). In this case we have a single time series for each bank with the daily returns of stock closing price spanning from January 2003 to May 2017. Thus, the graphical model obtained is representative of the partial correlations of the returns from 2003 to 2017.

Secondly, we calculate a different graphical model for each market day based on the data of the 90 previous days. Literally, we apply a moving window of length 90 days to the stock return time series and for every position of the moving window we fit a graphical model with the LoGo algorithm. Thus, for every market day, we obtain a network representative of the bank stocks returns correlations and market structure in the previous 90 days. All these networks can be seen as daily time series of graphical models from May 2003 to May 2017 (we start from May 2003 instead of January, due to the moving window lag). Both the mutual information and the transfer entropies are calculated for each edge of every graphical model with the distinction that for transfer entropy is calculated only for the edges that go from 1-day lagged nodes to contemporary ones. Calculating these measures only for the network edges is a great computational saving because it means computing around ca. 100 quantities per graphical model (the number of edges of our sparse LoGo inferred model) instead of calculating 2485 quantities ($(n^{2}-n)/2$, with *n* number of nodes). Then we aggregate the mutual information and transfer entropy time series both at the bank and system level. The system level aggregation produces measures that summarize the behaviour of the entire network and can be compared with the overall financial system stress indexes.

## 4. Results

**Figure 1:**
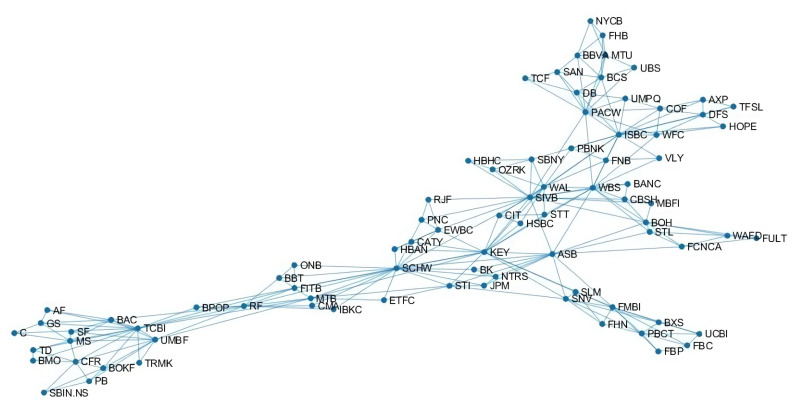
Network model inferred stock returns data for the period 2003–2017 by the LoGo algorithm.

Given the long time horizon considered in this paper, we would expect to see only some constant properties of the banks emerging from the graphical model structure, like characteristics connected to the bank dimension, business model, nationality. Interestingly from Figure 1 where the estimated network is represented using a spectral approach, we can see that many of the largest bank like C, BAC, GS, MS, TD are close to each other and connected by edges in the lower left corner of the network. At the same time, foreign banks like BBVA, BCS, DB, UBS are located together in the right top corner.

**Figure 2:**
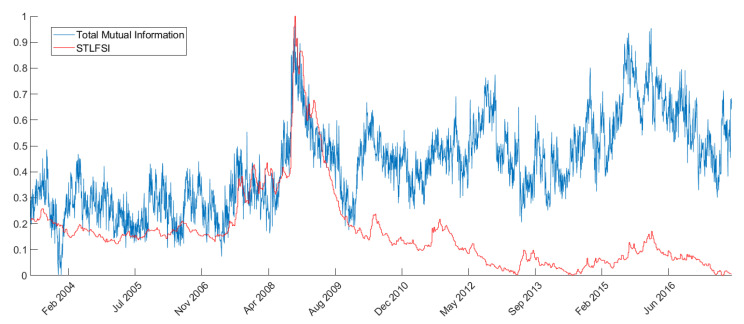
Total network mutual information vs STLFSI trends comparison over time. The values on the ordinates are rescaled so that both the Total network mutual information and the STLFSI have values ranging between 0 and 1 over the considered time-period.

As explained above, we aggregate the mutual information and transfer entropy time series both at the bank and system level. This produces measures that summarize the behaviour of the entire network and can be compared with the overall financial system stress indexes. For example, from Figure 2 it is possible to see how the network total mutual information resembles very closely (especially in the trends) the STLFSI. We can see from the figure that the trends are very similar and timely coincident, especially around the stress peaks registered during the 2008 financial crisis. This result is coherent with Reference [66] where the authors show that correlation spikes tend to predict or coincide with significant economic or market events, especially during the 2007–2008 financial crisis.

In Table 2 we report the results of the partial Granger causality test. From the results in Table 2, where the banks with at least two significant lags at α=0.05 are reported, we can see that the statistically significant banks comprehend both large banks like JPM, C, WFC, medium size banks like STL, ASB and small banks like NYCB. The list includes also large foreign banks like BBVA, HSBC and DB that have considerable activities in the US.

In Table 3 we report the most important banks according to the different analyzed time windows (2003–2006, 2007–2010, 2011–2017) and the measures of interest (Mutual Information, Transfer Entropy out., Transfer Entropy in.). Thus, given all the banks that have at least 2 significant lags at α=0.05 within a period and a specific bank measure, we select, by majority voting, those that appear the most. The rationale behind such choice relies on the fact that if a bank is particularly relevant in predicting one stress index, it is plausible to assume that it can give causality signals to all the stress indicators (STLFSI, Libor-OIS spread and CHF/USD rate return). In fact, from Table 4, we can observe that the number of statistically significant signals for stress index are evenly distributed among STLFSI, Libor-OIS spread and CHF/USD rate return. So, the most significant banks should have significant lags in predicting not only one stress index but possibly more. In Table 3 we have many of the largest US banks, in particular when the transfer entropy outflow is considered. This is reasonable because largest banks are more likely to influence the rest of the system and thus help in predicting it.

In the pre-crisis period ‘03-’06 we find less significant banks, as we can see from Table 5, especially when testing Granger causality for the STLFSI and Libor-OIS spread. This is expected since both the indexes have been widely adopted and regarded during and after the crisis. In particular, STLFSI has been developed after the crisis and backward calculated with the goal of being a good indicator for the crisis. Moreover both the indicators during the pre-crisis period were not subject to sudden and extensive spikes or changes thus is more difficult that a single bank stock is useful in predicting its behaviour.

During the crisis period ‘07-’10, there are more banks with statistically significant p-values in the partial Granger causality tests (49 statistically significant signals from Table 5) due to the greater correlation of the whole financial system, found also by other studies [66]. This is also in agreement with the fact that both the total mutual correlation and the transfer entropy of the network peak during the crisis. During the crisis is interesting to look at the banks whose transfer entropy outflow is most relevant in Granger causing the indexes. Note also that the transfer entropy exhibits less statistically significant signals than the mutual information as shown in Table 6. These banks in fact, are those whose influence on the rest of the system (transfer entropy outflow) is more useful to predict the stress indexes; they are mainly large banks (J.P. Morgan, Bank of America, Capital One Financial) that had an important role during the crisis.

In mid 2007, in response to the U.S. housing downturn, Capital One Financial (COF) posted great losses and announced that it would have cut 1900 jobs and shut down a wholesale mortgage unit it had acquired less than a year before.

Bank of America (BAC) is the second largest financial institution in the US and has been severely affected by the crisis. Several acquisitions in fact, had increased its exposition towards consumer credit and house mortgages. In 2005 it bought the credit card giant MBNA, in 2008 it acquired Countrywide Financial, the largest mortgage originator in America at the time and the troubled stockbroker Merrill Lynch. All of these businesses registered enormous losses during the crisis.

J.P. Morgan (JPM) has been a pivotal bank during the crisis in positive terms compared to the others. J.P. Morgan in fact, in the years prior to the crisis mostly avoided subprime mortgages, structured investment vehicles and collateralized debt obligations. When the subprime bubble triggered a massive deleveraging J.P. Morgan was mostly unharmed compared to its rivals. So J.P. Morgan was in such a good position, that it offered to take over Bear Sterns.

During the post-crisis period, ‘10-’17 we register less significant banks, in line with the intervention of the central banks whose policies have helped cooling down the financial system. Among the most relevant, we find both large banks like Wells Fargo (WFC) and Citigroup (C) and smaller institutes like Frost bank (CFR), Raymond James bank (RJF) and Sterling National bank (STL). The two large banks are bad performers among their peers. Wells Fargo while recovering from the crisis has witnessed a troubled post-crisis period studded with lawsuits and scandals that have undermined its reputation at the point that in 2018 the bank launched a marketing campaign called “Re-Established” to emphasize the company’s commitment to re-establish trust with stakeholders. Citigroup after the government bailout, has failed FED stress test in 2012 and 2014 and has seen a period of downsizing characterized by market exits, sell off and shutdown of different units. Instead the smaller statistically significant institutes (CFR, RJF and STL) are all characterized by an intense expansion and acquisition activity during the post crisis period.

It is important to note that there are some banks that are significant in more than one time period. For example, ASB, BANC and SAN are significant in all the three periods (‘03-’17), while SIVB, TCF, BBT, PNC are significant before and during the financial crisis (‘03-’10) and STL, SLM, JPM and STI (‘07-’17) are significant during and after the crisis. These banks comprehend both important hubs in the network model like ASB, SIVB and STL and more periferic nodes like TCF or SAN (see Figure 1).

## 5. Conclusions

In this paper we have presented two main contributions. Firstly, we have applied a recently presented graphical model inference methodology, LoGo, to the investigation of U.S. Banks stock returns to understand the network structure and evolution from 2003 to 2017. Thanks to the LoGo computational efficiency we have been able to estimate a separate graphical model for each market day and generate several time series of bank related measures computed from the network structure. Secondly, we have presented a way to leverage the graphical models information comparing the measures extracted from its structure with well known financial stress indexes and performed a causality analysis among them. To perform the causality analysis we resorted to the partial Granger causality method to take into account different control variables.

The inferred graphical models and the bank related measures extracted from them have shown to be an interesting tool for monitoring the U.S. bank system evolution. The network model is, in fact, useful for clustering groups of banks and see how these clusters evolve during time. The bank related measures extracted from the network have instead shown correlation with several financial stress indexes and to be linked in Granger causality terms to some of them acting as causing variables in the different time frames.

Our results show that in the pre-crisis period ‘03-’06 we have less significant banks, especially when testing Granger causality for the STLFSI and Libor-OIS spread. This is expected since both the indexes have been widely adopted and regarded during and after the crisis. During the crisis period ‘07-’10, there are more banks with statistically significant p-values in the partial Granger causality tests (49 statistically significant signals) due to the greater correlation of the whole financial system. In the post-crisis period, ‘10-’17 we register less significant banks, in line with the intervention of the central banks whose policies have helped cooling down the financial system. We interestingly noticed that both large and (relatively) small banks represent key actors in the financial system. This confirms the fully interconnectedess of the financial system where each player can represent a source of risk and contagion whatever the size.

Considering further research on this topic, it would be interesting to use other publicly available information on banks as well, like for example, bonds issued by banks or banks CDS. Bonds and CDS may capture different risk information more related to the bank default risk. In this case it would be necessary to handle different maturities in a proper way in order to obtain comparable variables.

On the other hand, other sources of data like banks liabilities would add a further level of information that would definitely enrich the analysis. Indeed, many analysis are used to explore the financial systems through the study of connections among financial institutions, employing banking liabilities and claims because such source of interconnection can clearly play a crucial role in propagating, absorbing or magnifying shocks.

However, the lack of bilateral data or publicly available ones, have hindered the systematical and comparative study of the characteristics of the international financial network. That said, scientific papers that had the chance to use such information have indubitable shown the relevance and the added value of the pair banks liabilities-network models [67,68]. Several interesting studies have been also conducted by adding further information that can be extracted by textual information derived from financial news [69,70] or social media [71]. A further development of our approach would then be to include textual data to further explain financial dynamics. Finally it would be of particular interest to merge the different obtained networks in a multilayer network model that could potentially capture different aspects of the banks risk.

## Figures and Tables

**Table 1:** List of the banks object of the study.

Bank	Ticker	Assets ($ bn)	Bank	Ticker	Assets ($ bn)
JPMorgan Chase Bank	JPM	2118	Banco Popular de Puerto Rico	BPOP	30
Wells Fargo Bank	WFC	1741	Frost Bank	CFR	30
Bank of America	BAC	1660	Synovus Bank	SNV	29
Citibank	C	1356	Associated Bank	ASB	29
U.S. Bank	USB	448	First Tennessee Bank	FHN	28
PNC Bank	PNC	358	Webster Bank	WBS	26
The Bank of New York Mellon	BK	300	Umpqua Bank	UMPQ	25
Capital One	COF	279	Commerce Bank	CBSH	25
TD Bank	TD	265	Whitney Bank	HBHC	23
State Street Bank	STT	252	Valley National Bank	VLY	22
Branch Banking and Trust Company	BBT	217	First National Bank of Pennsylvania	FNB	21
HSBC Bank USA	HSBC	204	Prosperity Bank	PB	21
SunTrust Bank	STI	200	Pacific Western Bank	PACW	21
Charles Schwab Bank	SCHW	165	TCF National Bank	TCF	21
Goldman Sachs Bank USA	GS	158	Iberiabank	IBKC	21
Fifth Third Bank	FITB	141	UMB Bank	UMBF	19
Morgan Stanley Bank	MS	127	MB Financial Bank	MBFI	19
Manufacturers and Traders Trust	MTB	126	Bank of the Ozarks	OZRK	18
Regions Bank	RF	124	Sallie Mae Bank	SLM	18
The Northern Trust Company	NTRS	120	Raymond James Bank	RJF	17
MUFG Union Bank	MTU	117	FirstBank	FBP	17
BMO Harris Bank	BMO	107	Bank of Hawaii	BOH	16
KeyBank	KEY	101	Washington Federal	WAFD	15
Huntington National Bank	HBAN	100	Astoria Bank	AF	15
Santander Bank	SAN	85	Old National Bank	ONB	15
Compass Bank	BBVA	85	BancorpSouth Bank	BXS	15
Comerica Bank	CMA	74	Flagstar Bank, FSB	FBC	14
Deutsche Bank Trust Company Americas	DB	55	Cathay Bank	CATY	14
American Express Bank	AXP	46	Sterling National Bank	STL	14
New York Community Bank	NYCB	46	Bank of Hope	HOPE	14
Silicon Valley Bank	SIVB	43	Trustmark National Bank	TRMK	13
People’s United Bank	PBCT	40	First Midwest Bank	FMBI	11
E*TRADE Bank	ETFC	36	Stifel Bank and Trust	SF	11
East West Bank	EWBC	33	Banc of California	BANC	11
First-Citizens Bank & Trust Company	FCNCA	33	Fulton Bank	FULT	11
BOK Financial	BOKF	33	United Community Bank	UCBI	10
Barclays Bank Delaware	BCS	31	State Bank of India	SBIN	10

**Table 2:** Partial Granger causality results: causality test from bank level measures to financial stress indexes; statistically significant banks have at least two significant lags at α = 0.05.

Period	Bank Level Measure	Financial Stress Index	Statistically Significant Banks
‘03-’06	Mutual Information	CHF/USD returns	ASB, CBSH, PACW, TCF, UMPQ
‘03-’06	Mutual Information	Libor-OIS spread	BBT, PNC
‘03-’06	Mutual Information	STLFSI	AF, UMPQ
‘03-’06	Transfer Entropy out.	CHF/USD returns	BANC, IBKC, OZRK
‘03-’06	Transfer Entropy out.	Libor-OIS spread	AF, BANC, PBCT
‘03-’06	Transfer Entropy out.	STLFSI	BOKF, FNB, SAN, SCHW, SIVB
‘03-’06	Transfer Entropy in.	CHF/USD returns	FBP, IBKC, NYCB, RF
‘03-’06	Transfer Entropy in.	Libor-OIS spread	FCNCA, FITB, PACW, SIVB
‘03-’06	Transfer Entropy in.	STLFSI	BMO, FITB, FULT
‘07-’10	Mutual Information	CHF/USD returns	AXP, BK, DB, SBIN, SIVB, UCBI, WBS
‘07-’10	Mutual Information	Libor-OIS spread	CMA, DB, FHN, FITB, HBAN, SAN, TD, UCBI
‘07-’10	Mutual Information	STLFSI	BANC, BK, PNC, STL
‘07-’10	Transfer Entropy out.	CHF/USD returns	GS, MTB, SLM
‘07-’10	Transfer Entropy out.	Libor-OIS spread	ASB, BAC, BBT, BBVA, COF, JPM, TCF
‘07-’10	Transfer Entropy out.	STLFSI	BAC, BANC, BCS, COF, JPM, STI
‘07-’10	Transfer Entropy in.	CHF/USD returns	BBT, BXS, KEY, SBIN, WFC
‘07-’10	Transfer Entropy in.	Libor-OIS spread	HBAN, HSBC, PBCT, SBIN, WBS
‘07-’10	Transfer Entropy in.	STLFSI	AF, BANC, SBIN, WBS
’11-’17	Mutual Information	CHF/USD returns	BOKF, C, CFR, FBC, JPM, MTU, RF, RJF, STL
’11-’17	Mutual Information	Libor-OIS spread	BANC, C, CFR, RJF, STL
’11-’17	Mutual Information	STLFSI	C, CFR, RJF, SAN, SLM, STI, STL, VLY
’11-’17	Transfer Entropy out.	CHF/USD returns	
’11-’17	Transfer Entropy out.	Libor-OIS spread	ASB, BOKF
’11-’17	Transfer Entropy out.	STLFSI	NYCB, SF, UMBF
’11-’17	Transfer Entropy in.	CHF/USD returns	
’11-’17	Transfer Entropy in.	Libor-OIS spread	BBVA, TD, WFC
’11-’17	Transfer Entropy in.	STLFSI	CFR, OZRK, WFC

**Table 3:** Most significant banks: list of the banks that appear more times as statistically significant banks in Table 2 given a reference period and a bank level measure.

Period	Bank Level Measure	Mostly Significant Banks
2003–2006	Mutual Information	
2003–2006	Transfer Entropy outflow	BANC
2003–2006	Transfer Entropy inflow	FITB
2007–2010	Mutual Information	BK, DB
2007–2010	Transfer Entropy outflow	BAC, COF, JPM
2007–2010	Transfer Entropy inflow	SBIN
2011–2017	Mutual Information	C, CFR, RJF, STL
2011–2017	Transfer Entropy outflow	
2011–2017	Transfer Entropy inflow	WFC

**Table 4:** Number of statistically significant banks signals per stress index: for each stress index is reported the number of statistically significant signals from Table 2; statistically significant banks have at least two significant lags at α = 0.05.

Stress Index	Num. of Stat. Signif. Bank Signals
STLFSI	38
Libor-OIS spread	39
CHF/USD	36

**Table 5:** Number of statistically significant banks signals per period: for each time period is reported the number of statistically significant signals from Table 2; statistically significant banks have at least two significant lags at α = 0.05.

Period	Num. of Stat. Signif. Bank Signals
2003–2006	31
2007–2010	49
2011–2017	33

**Table 6:** Number of statistically significant banks signals per information theory measure: for each measure is reported the number of statistically significant signals from Table 2; statistically significant banks have at least two significant lags at α = 0.05.

Information Theory Measure	Num. of Stat. Signif. Bank Signals
Mutual information	50
Transfer entropy inflow	31
Transfer entropy outflow	32

## Appendix A

**Algorithm A1:** Algorithm A1 to compute the Mutual Information timeseries of each bank with the rest of banking system from the fitted Graphical Gaussian Models

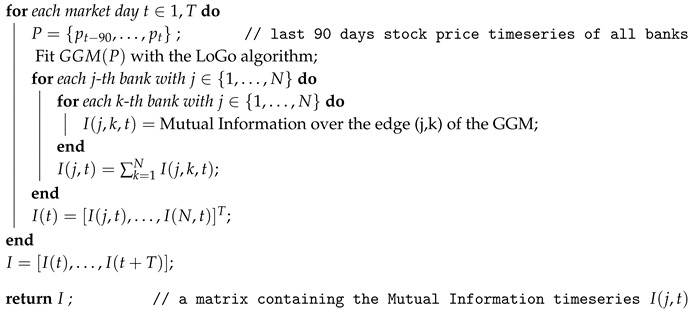

**Algorithm A2:** Algorithm A2 to compute the Transfer Entropy Outflow timeseries of each bank towards the rest of banking system from the fitted Graphical Gaussian Models

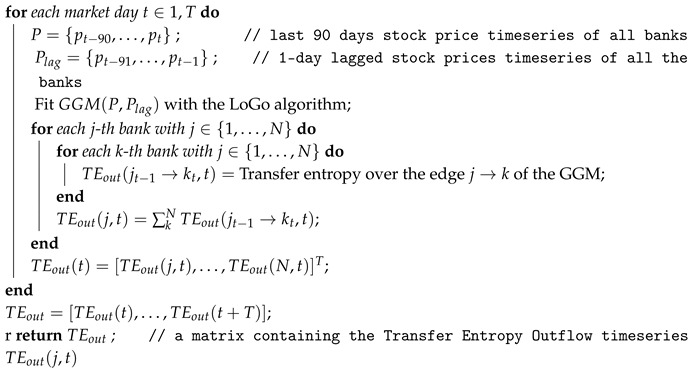

**Algorithm A3:** Algorithm A3 to compute the Transfer Entropy Inflow timeseries of each bank from the rest of banking system from the fitted Graphical Gaussian Models

## References

[1] Ozgöde O. The Emergence of Systemic Financial Risk: From Structural Adjustment (Back) to Vulnerability Reduction. www.limn.it.

[2] Caccioli F., Barucca P., Kobayashi T. (2018). Network models of financial systemic risk: A review. J. Comput. Soc. Sci..

[3] Tungsong S., Caccioli F., Aste T. (2018). Relation between regional uncertainty spillovers in the global banking system. J. Netw. Theory Financ..

[4] Acharya V.V., Pedersen L.H., Philippon T., Richardson M. (2017). Measuring Systemic Risk. Rev. Financ. Stud..

[5] Adrian T., Brunnermeier M.K. (2016). CoVaR. Am. Econ. Rev..

[6] Acharya V., Engle R., Richardson M. (2012). Capital shortfall: A new approach to ranking and regulating systemic risks. Am. Econ. Rev..

[7] Brownlees C.T., Engle R.F. (2011). Volatility, Correlation and Tails for Systemic Risk Measurement. SSRN Electron. J..

[8] Huang X., Zhou H., Zhu H. (2012). Systemic risk contribution. J. Financ. Serv. Res..

[9] Cao Z. (2013). Multi-CoVaR and Shapley Value: A Systemic Risk Measure. Work. Pap. Banq. Fr.-Dsf-Smf.

[10] Banulescu G.D., Dumitrescu E.I. (2014). Which are the SIFIs? A component expected shortfall approach to systemic risk. J. Bank Financ..

[11] Calabrese R., Giudici P. (2015). Estimating bank default with generalised extreme value models. J. Oper. Res. Soc..

[12] Bardoscia M., Battiston S., Caccioli F., Caldarelli G. (2015). DebtRank: A microscopic foundation for shock propagation. PLoS ONE.

[13] Bardoscia M., Battiston S., Caccioli F., Caldarelli G. (2017). Pathways towards instability in financial networks. Nat. Commun..

[14] Caccioli F., Shrestha M., Moore C., Farmer J.D. (2014). Stability analysis of financial contagion due to overlapping portfolios. J. Bank Financ..

[15] Cerchiello P., Giudici P., Nicola G. (2017). Twitter data models for bank risk contagion. Neurocomputing.

[16] Billio M., Getmansky M., Lo A., Pelizzon L. (2012). Econometric measures of connectedness and systemic risk in the finance and insurance sector. J. Financ. Econ..

[17] Hautsch N., Schaumburg J., Schienle M. (2014). Forecasting Systemic Impact in Financial Networks. Int. J. Forecast..

[18] Peltonen T.A., Piloiu A., Sarlin P. (2015). Network Linkages to Predict Bank Distress.

[19] Diebold F.X., Yilmaz K. (2014). On the Network Topology of Variance Decompositions: Measuring the Connectedness of Financial Firms. J. Econom..

[20] Mantegna R.N. (1999). Hierarchical Structure in Financial Markets. Eur. Phys. J. B.

[21] Aste T., Di Matteo T., Tumminello M., Mantegna R.N. (2005). Correlation filtering in financial time series’. J. Noise Fluct. Econophys. Financ..

[22] Tumminello M., Aste T., Di Matteo T., Mantegna R.N. (2005). A tool for filtering information in complex systems. Proc. Natl. Acad. Sci. USA.

[23] Cerchiello P., Giudici P. (2016). Conditional graphical models for systemic risk estimation. Expert Syst. Appl..

[24] Giudici P., Spelta A. (2016). Graphical network models for international financial flows. J. Bus. Econom. Stat..

[25] Barigozzi M., Brownlees C. (2019). Nets: Network Estimation for Time Series. J. Appl. Econ..

[26] Barfuss W., Massara G.P., Di Matteo T., Aste T. (2016). Parsimonious modeling with information filtering networks. Phys. Rev. E.

[27] Guo S., Seth A.K., Kendrick K.M., Zhou C., Feng J. (2008). Partial Granger causality–eliminating exogenous inputs and latent variables. J. Neurosci. Methods.

[28] Pearl J. (1999). Causality: Models, Reasoning, and Inference.

[29] Roelstraete B., Yves R. (2011). FIAR: An R Package for Analyzing Functional Integration in the Brain. J. Stat. Softw..

[30] Hyndman R., Athanasopoulos G., Bergmeir C., Caceres G., Chhay L., O’Hara-Wild M., Petropoulos F., Razbash S., Wang E., Yasmeen F. Forecast: Forecasting Functions for Time Series and Linear Models. http://pkg.robjhyndman.com/forecast.

[31] Hyndman R.J., Khandakar Y. (2008). Automatic time series forecasting: The forecast package for R. J. Stat. Softw..

[32] Frank O., Strauss D. (1986). Markov graphs. J. Am. Stat. Assoc..

[33] Holland P.W., Leinhardt S. (1981). An exponential family of probability distributions for directed graphs. J. Am. Stat. Assoc..

[34] Nowicki K., Snijders T.A. (2001). Estimation and prediction for stochastic blockstructures. J. Am. Stat. Assoc..

[35] Wang Y.J., Wong G.Y. (1987). Stochastic blockmodels for directed graphs. J. Am. Stat. Assoc..

[36] Handcock M.S., Raftery A.E., Tantrum J.M. (2007). Model-based clustering for social networks. J. R. Stat. Soc. Ser. A Stat. Soc..

[37] Hoff P.D., Raftery A.E., Handcock M.S. (2002). Latent space approaches to social network analysis. J. Am. Stat. Assoc..

[38] Goldenberg A., Zheng A.X., Fienberg S.E., Airoldi E.M. (2010). A survey of statistical network models. Found. Trends Mach. Learn..

[39] Kolaczyk E.D. (2009). Statistical Analysis of Network Data: Methods and Models.

[40] Kolaczyk E.D., Csárdi G. (2014). Statistical Analysis of Network Data with R.

[41] Snijders T.A. (2011). Statistical models for social networks. Annu. Rev. Sociol..

[42] Ahelegbey D.F., Billio M., Casarin R. (2016). Bayesian graphical models for structural vector autoregressive processes. J. Appl. Econ..

[43] Heckerman D., Geiger D., Chickering D.M. (1995). Learning Bayesian networks: The combination of knowledge and statistical data. Mach. Learn..

[44] Carvalho C.M., West M. (2007). Dynamic matrix-variate graphical models. Bayesian Anal..

[45] Carvalho C.M., Massam H., West M. (2007). Simulation of hyper-inverse Wishart distributions in graphical models. Biometrika.

[46] Dahlhaus R., Eichler M. (2003). Causality and graphical models for time series. Oxf. Stat. Sci. Ser..

[47] Eichler M. (2007). Granger causality and path diagrams formultivariate time series. J. Econom..

[48] Zou C., Feng J. (2009). Granger causality vs. dynamic Bayesian network inference:a comparative study. BMC Bioinform..

[49] Diks C., Panchenko V. (2005). A note on the Hiemstra–Jones test for Granger noncausality. Stud. Nonlinear Dyn. Econ..

[50] Hoover K.D. (2001). Causality in Macroeconomics.

[51] Lauritzen S.L. (1996). Graphical Models.

[52] Massara G.P., DiMatteo T., Aste T. (2016). Network Filtering for Big Data: Triangulated Maximally Filtered Graph. J. Complex Netw..

[53] MacKay D.J.C. (2003). Information Theory, Inference, and Learning Algorithms.

[54] Vicente R., Wibral M., Lindner M., Pipa G. (2011). Transfer entropy—A model-free measure of effective connectivity for the neurosciences. J. Comput. Neurosci..

[55] Schreiber T. (2000). Measuring Information Transfer. Phys. Rev. Lett..

[56] Wyner A.D. (1978). A definition of conditional mutual information for arbitrary ensembles. Inf. Control..

[57] Hlaváčková-Schindler K., Palus M., Vejmelka M., Bhattacharya J. (2007). Causality detection based on information-theoretic approaches in time series analysis. Phys. Rep..

[58] Granger C.W.J. (1969). Investigating Causal Relations by Econometric Models and Cross-spectral Methods. Econometrica.

[59] Sims C. (1972). Money, Income, and Causality. Am. Econ. Rev..

[60] Kendall M., Stuart A. (1979). The Advanced Theory of Statistics: Inference and Relationship.

[61] Roelstraete B., Yves R. (2012). Does Partial Granger Causality Really Eliminate the Influence of Exogenous Inputs and Latent Variables?. J. Neurosci. Methods.

[62] Federal Reserve Bank of St. Louis St. Louis Fed Financial Stress Index. Retrieved from FRED. https://fred.stlouisfed.org/series/STLFSI.

[63] Kevin L., Kliesen D., Smith C. (2010). Measuring Financial Market Stress. St. Louis Fed Econ. Synopses.

[64] Sengupta R., Tam Y. (2008). The LIBOR-OIS spread as a summary indicator. Monetary Trends.

[65] Deutsche Bundesbank (2014). Exchange Rates and Financial Stress.

[66] Dilip K., Patro M.Q., Xian S. (2013). A simple indicator of systemic risk. J. Financ. Stab..

[67] Giudici P., Spelta A., Sarlin P. (2000). The interconnected nature of financial systems: Direct and common exposures. J. Ban. Fin..

[68] Quadrini V. (2017). Bank liabilities channel. J. Monet. Econ..

[69] Cerchiello P., Nicola G. (2018). Assessing News Contagion in Finance. Econometrics.

[70] Cerchiello P., Giudici P. (2016). Big data analysis for financial risk management. J. Big Data.

[71] Ranco G., Aleksovski D., Caldarelli G., Grčar M., Mozetič I. (2015). The Effects of Twitter Sentiment on Stock Price Returns. PLoS ONE.

